# Adolescents' Lived Experiences of Social Media on Perceived Body Image: A Qualitative Synthesis of Evidence

**DOI:** 10.1111/inm.70379

**Published:** 2026-10-22

**Authors:** Isobel Whibley, Mark Pearson

**Affiliations:** ^1^ University of Nottingham Nottingham UK; ^2^ University of Lincoln Lincoln UK

**Keywords:** adolescents, body image, mental health, qualitative, qualitative evidence synthesis, social media (SM)

## Abstract

Adolescence is a transformational period in terms of the formation of identity and emotional development. Some quantitative research studies have attributed the growing instances of body dissatisfaction and disordered eating behaviours in adolescents to the harmful standards perpetuated by social media. However, limited qualitative research has been conducted to consider adolescents' perspectives and lived experiences. This literature review aims to explore the lived experiences and perspectives of adolescents who use social media, and how social media is perceived as affecting body image. CINAHL, PubMed and MEDLINE were searched from 2013 to 2025 for qualitative studies investigating the impact of social media on adolescents' perceived body image. The quality of the included papers was assessed using the Critical Appraisal Skills Programme (CASP) tool for qualitative research. Seven studies were included in the review. Data were analysed using thematic analysis, which generated the themes of Identity and self‐perception, gendered experiences and Protective roles and external influences. This review found that participants in the included studies perceived unattainable beauty standards and societal pressures propagated by social media caused increased body dissatisfaction, self‐comparison and self‐objectification. For some participants, this consequently incurred disordered eating behaviours. It was concluded that adolescents frequently perceived social media use as having an adverse influence on their body image. Parents and school educators are key protective factors in mitigating this. Further research should be conducted to ascertain the potential for compassion‐focused interventions in school curricula and body‐positive content on social media, to improve adolescent body image and enhance media literacy.

## 1 Introduction

The psychological impact of social media (SM) engagement during adolescence represents a relatively novel area of research (Holland and Tiggeman 2016). While some studies have identified potential benefits, such as enhanced social connectedness and improved self‐esteem (Valkenburg and Peter 2009), a growing body of evidence has highlighted significant concerns. Specifically, SM use in adolescence has been associated with heightened levels of body dissatisfaction, appearance comparisons and the development or exacerbation of disordered eating behaviours (Sidani et al. 2016; Salazar 2017). While there is an emerging body of literature exploring the harmful impacts of SM use within the adolescent population, it is clear that further research is needed in this area to reflect the growing popularity of SM sites and the wide public discourse surrounding them.

Fusar‐Poli et al. (2020) defined mental health as a ‘state of well‐being’ that is characterised by stable self‐perceptions, constructive coping mechanisms for external stressors, and normal emotional development. Research suggests that the majority of determinants of mental health are encountered in the adolescent stage of development, with diet cultures, pressures to conform to beauty standards, and adverse social interactions with peers being some of the leading risk factors for the development of mental health conditions in adolescence (World Health Organization 2022; Lin and Guo 2024). Adolescents are commonly exposed to these risk factors on SM, which can be defined as platforms that promote interpersonal interaction through the distribution of users' own media and sharing reactions to others' content (Carr and Hayes 2015). Cross‐culturally, adolescents have consistently reported that accessing SM gives rise to these factors, with one Australian teen reporting that accessing a ‘second world’ on SM with increased frequency caused the bullying that she was experiencing at school to become a ‘round‐the‐clock issue’, as her peers at school could contact her wherever she was (Lavoipierre 2024). She also reported that seeing thin bodies being idolised on SM platforms such as TikTok or Instagram caused her mental health to decline, specifically regarding body image (Lavoipierre 2024).

Given that there are 5.24 billion active SM users globally (Petrosyan 2025), it is interesting to note that levels of body dissatisfaction and cyberbullying within the adolescent demographic are rising in correlation with this growing popularity (Salazar 2017). The adverse effects on mental health also correlate with augmented rates of referrals to Child and Adolescent Mental Health Services (CAMHS) in the UK, and the prominence of body dissatisfaction in this age group. The youth mental health charity YoungMinds has indicated the scale of this issue by ‘calling for further government action’ as a result of their analysis of NHS Digital data, reporting that the number of under‐18s currently open to CAMHS has risen by 53% since 2019, only 6 years ago (YoungMinds 2023). Further research revealed that the exponential rise in adolescents engaging in these behaviours is consistent with Ofcom's (2011) report, finding a 256% growth in time spent online in the same participant group over 6 years. These trends appear to coincide with increasing screen time and rising numbers of adolescents seeking mental health support.

Adolescence involves physical and emotional development, as well as the formation of an individual identity. Within this, physical appearance becomes of increased importance (Salomon and Brown 2018), and how young people view themselves during this time is related to their self‐perceived body image. This is defined by Grogan (2008), as how one evaluates one's own body, which is based on a personally constructed perspective, and is influenced by interactions with others. Body dissatisfaction develops due to ‘distortion of the body image’ (Cohen et al. 2017) and internalisation of idealistic beauty standards (Sharp and Gerrard 2022), which causes increased body surveillance and is a common precursor to disordered eating behaviour (Rodgers and Melioli 2016). Researchers argue this occurs through social comparison theory, when young people, particularly women, compare their physical appearance to that of others (Festinger 1954; Van den Berg et al. 2002). It is clear that there is a link between SM use and negative body image, which leads to the potential development of disordered eating.

The mental health and well‐being of the youth is a global crisis (Patton et al. 2016), with epidemiological studies describing the ‘peak onset’ of most mental disorders being 15 years of age, with 63%–75% occurring during the ‘transitional period from childhood to adulthood’ (Solmi et al. 2022; McGorry et al. 2024). The Lancet Psychiatry Commission on Youth Mental Health (McGorry et al. 2024) has additionally identified the ‘alarming’ global megatrends that are the driving forces of psychological distress and mental health issues in young people, with ‘the rise in social media’ being mentioned at the forefront, alongside global (environmental, political and financial) instability and intergenerational inequality. It could be argued, based on the accumulating research evidence, that adolescent mental health is becoming a global issue and researchers are attributing this partly to SM's influence on the younger generation.

A previous qualitative systematic review undertaken by Popat and Tarrant (2023) explored adolescents' perspectives on social media and mental health and well‐being. The results identified ‘appearance concerns and body ideals’ as a theme within the literature, with adolescents highlighting the perceived importance of ‘perfect’ photos and the social validation of receiving ‘likes’. However, due to the wider focus of this review on mental health and well‐being the impact on body image was not examined in sufficient depth. Therefore, a knowledge gap remains in relation to understanding adolescents' complex lived experiences and perceptions of social media use in relation to body image. This review addresses this remaining knowledge gap.

## 2 Background and Rationale for the Review

The World Health Organisation emphasised the notability of promoting a wider understanding of adolescent mental health in a 2021 Newsroom article. It was disclosed that one in seven young people experiences a mental disorder in their teen years, with depression, anxiety and eating disorders being some of the leading causes of illness and disability in this age group (World Health Organization 2021). Further research showed that 31% of 16–24‐year‐olds reported experiencing anxiety and depression in 2017–2018, which had increased by 26% from 2016, and a further 26% from 2011, clearly showing an accelerated trend of growth (Office for National Statistics 2020). These results also correlate with a follow‐up on the 2017 NHS survey regarding the mental health of young people in England, which showed a similar increase in the rates of young people with mental disorders from 10.1% in 2017 to 25.7% in 2022 (NHS Digital 2022). There is a substantial body of evidence supporting the increasing prevalence of mental disorders in adolescents, evidenced by the wealth of statistics on the subject.

Perceptions of body image during adolescence are complex and multifactorial, including components such as acceptance of one's body, normalisation of differences and security with oneself (Tort‐Nasarre et al. 2023). The relationship between SM and body image is supported by a large literature base, with researchers suggesting that usage of visual‐based platforms such as Instagram or TikTok and the domination of the ‘thin‐ideal’ in this media is causally linked to the development of body dissatisfaction and disordered eating symptoms in young people (Sidani et al. 2016). The ‘thin‐ideal’ describes an unattainably thin figure that is commonly idolised in the media (Hawkins et al. 2004). This is estimated by researchers to be typically 15% below the average weight of women, making it biogenetically difficult for most women consuming this media, to achieve it (Attie and Brooks‐Gunn 1989). Social learning theory (Brown 2002, 42–45) states that young people who view this content start to internalise the body standards portrayed as normal, expected, and necessary for attractiveness (Grabe et al. 2008, 460–476). Further research indicates that this leads to the thin ideal being internalised by the audience of the content creator, through self‐discrepancy theory (Higgins 1987; McLean et al. 2015; Wick and Keel 2020). This theory describes a self‐perceived ‘discrepancy’ between the actual self and the ideal self, which is theorised to incur body dissatisfaction and psychological distress (Higgins 1987; Hargreaves and Tiggemann 2004) and has been shown to be a precursor to disordered eating behaviours (Stice and Shaw 2002). Considering this perspective, it could be argued that the growing popularity of SM and the numerous users utilising editing tools for their content is contributing to heightened levels of body dissatisfaction among young people. When an unrealistic body standard is promoted and popularised on SM, and young people engage with it, it arguably leads to diminished feelings of self‐worth, increased body dissatisfaction, and disordered eating behaviours.

Despite the presence of research examining the relationship between mental health and SM, a significant majority of studies, including those referenced above, concentrate on quantitatively correlating this relationship, with relatively few exploring the lived experiences of adolescents who engage with SM. This creates a gap for qualitative exploration of the potential consequences that increased SM engagement may have on adolescent mental health, particularly pertaining to body image. This underscores the heightened importance of investigating why SM negatively impacts mental health, as well as considering the effects this could have on this age group as they develop.

An introductory review of some of the statistics surrounding adolescent mental health and body image shows a strong connection to the increased use of SM in recent years. Consequently, the mental health of young people is declining, and addressing their mental health needs is becoming an urgent matter. According to NHS digital data, over 1.2 million young individuals were in contact with CAMHS in 2022 (YoungMinds 2023), highlighting the necessity for prioritising ‘service improvement in the NHS’ (Maddison et al. 2023). To manage the growing number of children using these services, more funding and government focus on CAMHS is essential. Increased resources would lead to hiring and training new staff, enabling a higher volume of appointments and shorter wait times. This would also address the challenge highlighted by Griffiths et al. (2001), where nurses reported facing difficulties integrating research into their practice, due to limited time and resources. Reducing workloads can motivate nurses to incorporate current research into their practices, aligning with section 6 of the NMC code, which states to ‘always practice in line with the best available evidence’, and to ‘maintain the knowledge and skills’ needed for contemporary practice (Nursing and Midwifery Council 2018).

Additionally, encouraging the two‐thirds of adolescents with mental health concerns who do not seek help (NHS Digital 2022) could be enhanced through studies on their lived experiences on SM, as outlined in this dissertation. This research could improve practices by increasing CAMHS visibility on social platforms and providing content that is accessible and relevant to their age group. Such engagement would promote resources that resonate with young individuals experiencing online challenges. However, for this strategy to be effective, the issues raised by Griffiths et al. must be addressed; CAMHS will struggle to handle increased engagement if challenges surrounding funding and wait times persist.

### 2.1 Aim

To explore adolescents' lived experiences and perceptions of social media use in relation to body image.

## 3 Methods

### 3.1 Research Question

To produce a comprehensive literature review, a clearly focused research question must be formulated (Aveyard 2023). Given the experiential focus of this research, the SPIDER framework was utilised to generate the research question as shown in Table 1.

**TABLE 1:** The SPIDER tool applied to the proposed research question.

S, sample	Adolescent social media users.
PI, phenomenon of interest	The impact of social media on adolescents' perceived body image.
D, design	Published works using a qualitative research design.
E, evaluation	Experiences and perceptions of the links between social media and adolescent body image.
R, research type	Primary qualitative studies that are peer‐reviewed.

The final research question developed for this research is ‘What are adolescents' lived experiences and perceptions of social media use in relation to their body image?’

### 3.2 Inclusion and Exclusion Criteria

Stern et al. (2014) argue that clear inclusion and exclusion criteria define the boundaries of the review by specifying which papers should be included and which are irrelevant to the subject under investigation. All inclusion and exclusion criteria are presented in Table 2. Quantitative studies were excluded due to the lived experience focus of the review (Tai and Ajjawi 2016). Adolescents are defined as young people aged between 12 and 18 years. No exclusions were made based on the geographical location of the studies.

**TABLE 2:** Inclusion and exclusion criteria.

Inclusion criteria	Exclusion criteria
Adolescents aged 12–18	Non‐adolescents, under 12 s and over 18 s
Studies published in the English language	Not published in the English language
Primary research	Secondary, non‐empirical research; articles, reviews, meta‐analyses
Studies published from 2013‐present	Studies published before 2013
Peer‐reviewed papers	Papers that are not peer‐reviewed
Qualitative studies, or mixed‐method design studies with a prominent qualitative focus	Quantitative studies, or mixed‐method design studies with a prominent quantitative focus
Research that does explore the impacts of social media on adolescents' body image	Research that does not focus on the impacts of social media on adolescents' body image
Studies containing interviews, focus groups or questionnaires in the research design	Studies containing experiments or surveys eliciting numerical data in the research design

### 3.3 Search Strategy

To achieve a comprehensive scope of research with a medical focus during the search process, advanced databases such as CINAHL, MEDLINE and PubMed were utilised to procure the largest number of relevant publications related to the subject area, ensuring that no pertinent studies were omitted. The National Institute of Health and Care Excellence (NICE) methods manual recommends the utilisation of multiple databases during the search process to produce a systematic and reproducible review of the literature (NICE 2022).

The terms generated from the SPIDER tool were used to translate the research question into searchable keywords (see Table 3), to access the most relevant papers on each database (Grewal et al. 2016). Boolean operators such as “AND” and “OR” were used to further focus the search (Fink 2020). Truncation (*) was also used to allow papers that contain different formats of the keywords, such as adolescents or adolescence, to be retrieved (Aromataris and Riitano 2014).

**TABLE 3:** Search strategies employed to yield a comprehensive scope of relevant literature.

Keywords	Boolean operators	Related terms	Truncation	
Social media	OR	“Social Networking Sites” OR “Digital media” OR “Social networks” OR “Online” OR “Internet”	N/A	“AND”
Adolescents	OR	“Teenagers” OR “Young adults” OR “Teen” OR “Youth” OR “Young people” OR “Adolescence”	Adolescen* OR Teen*	“AND”
Body Image	OR	“Self‐esteem” OR “Self‐image” OR “Body dissatisfaction”	N/A	“AND”
Experiences	OR	“Impact” OR “Qualitative” OR “Interviews”	N/A	

The data were reported and evaluated systematically in keeping with the PRISMA 2020 guidelines (Page et al. 2021) and adapted from the QUORUM checklist to prevent inadequate syntheses of data (Moher 2019).

### 3.4 Appraising the Literature

After selecting and screening the chosen papers for this extended literature review, each paper was critically appraised using the Critical Appraisal Skills Programme for Qualitative Research (CASP) (2022) tool. The CASP tool is the most frequently used instrument for critical appraisal in healthcare research and was therefore deemed the most suitable framework for the context of this review. The CASP tool considers two questions related to screening, and eight regarding the quality of the data collection method, consequences of results and how results can be extrapolated to a wider population (Hopkins et al. 2024).

Use of the CASP tool informed both the synthesis and interpretation of the findings. Studies with greater methodological rigour were given greater weight when identifying emergent themes and drawing conclusions, whereas findings from studies with specific methodological limitations, identified through the CASP assessment, were considered more cautiously (Long et al. 2020). For example, several studies reported gender differences in relation to the lived experiences of SM. However, studies such as Papageorgiou et al. (2022b), which only included female participants, were interpreted cautiously when developing a theme in relation to gender. Due to the focus on lived experience, specific consideration was also given to the sites of recruitment, with several studies primarily recruiting from privately funded schools, such as Burnette et al. (2017) or sites which were likely to yield predominantly older adolescents (Ando et al. 2021). Consequently, these studies were interpreted cautiously when developing themes intended to reflect wider adolescent populations.

See Appendix B for full details of CASP evaluation.

### 3.5 Data Analysis

Following comprehensive critical appraisal of the included papers, an inductive thematic analysis was conducted using Braun and Clarke's (2022) six‐phase framework, involving familiarisation with the data, coding, theme development and refinement and production of the final report. One member of the research team independently generated the initial codes and identified patterns across the data. The initial codes and patterns were then developed into themes based on shared concepts across the data. The initial themes were then examined and refined through a series of meetings with the secondary member of the research team, during which alternative interpretations were discussed and themes were refined to support coherence and maintain close alignment with the included studies.

Throughout the process of data analysis, the research team engaged in personal and disciplinary reflexive discussions (Wilkinson 1988). As both members of the research team have experience working within mental health services and with young people, these discussions enabled the research team to remain aware of their own subjectivity and to engage critically with their potential assumptions in relation to the data. In particular, the researchers reflected on how their professional experiences and prior expectations may have influenced interpretation of the findings. Reflexive discussion was integrated throughout the coding, theme development and refinement stages to critically examine potential biases and explore alternative interpretations of the data. This strengthened the credibility of the analysis by supporting the development of themes grounded in the findings of the included studies rather than shaped by researchers' preconceptions.

## 4 Findings

The initial search identified 281 papers. After duplicates and studies not meeting the inclusion criteria were excluded, seven studies remained. This process can be appropriately summarised using a PRISMA flow diagram (Figure 1).

**FIGURE 1:**
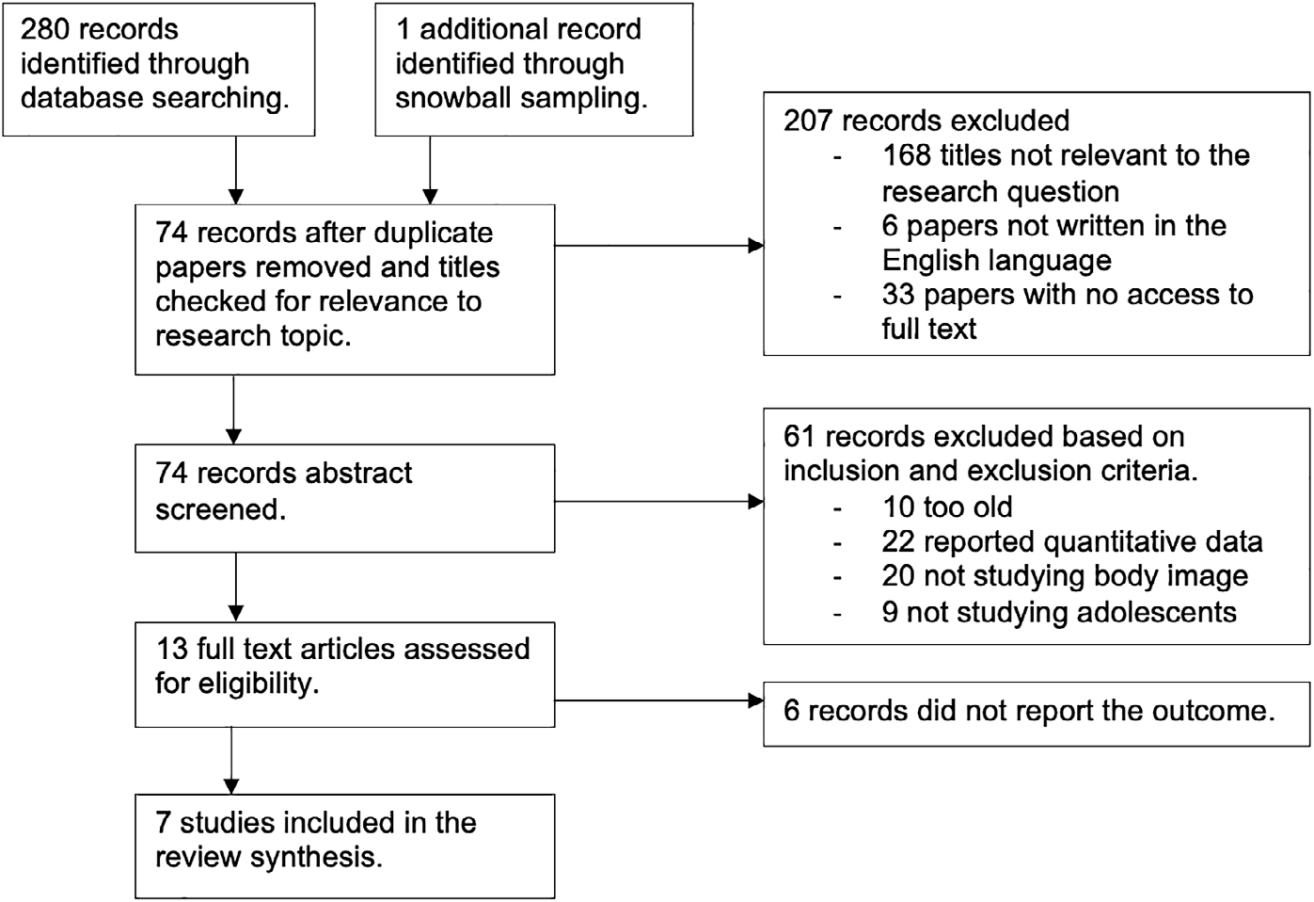
PRISMA diagram.

To explore the research question, ‘What are adolescents' lived experiences of the impact of social media on perceived body image?’, seven studies were included in the review (Papageorgiou et al. 2022a; Burnette et al. 2017; Spurr et al. 2013; Ando et al. 2021; Papageorgiou et al. 2022b; Artigues‐Barberà et al. 2025; Mahon and Hevey 2021). An overview of the included studies can be found in the Matrix (Appendix A). All seven studies presented qualitative data. Among the seven studies, three utilised focus groups as their method for data collection, and four used semi‐structured interviews. All included papers utilised thematic analysis to present their findings. Per the inclusion criteria, there were no geographical restrictions on the papers included in the review due to the global and cross‐cultural relevance of the lived experience being explored. From this, included studies were conducted in Japan, the United States, Australia, Canada, Ireland and Spain. All studies sought to explore adolescents' perceptions of how SM has affected their perceived body image, as indicated by the title and research question of this review.

The quality of the included studies was assessed using the Critical Appraisal Skills Programme (2022) critical appraisal tool for qualitative studies, which is demonstrated in Appendix B. Thematic analysis was employed to discuss the findings of each included study. Seven themes were identified in the included studies. These were subsequently developed into three overarching themes to present the most important and commonly occurring themes for discussion. These include ‘Identity and self‐perception’, ‘Gendered experiences’ and ‘Protective roles and external influences’. Appendix C outlines the identification of each theme in the included studies, which will be explored further in this chapter.

### 4.1 Theme 1: Identity and Self‐Perception

Across all the included studies, adolescents perceived SM use as influencing their identity and self‐perception regarding body image (Papageorgiou et al. 2022a, 2022b; Burnette et al. 2017; Spurr et al. 2013; Ando et al. 2021; Artigues‐Barberà et al. 2025; Mahon and Hevey 2021). From identifying commonly occurring findings, three sub‐themes were developed. These are self‐comparison, societal standards and pressures and validation.

### 4.2 Self‐Comparison

Of the seven studies included in this review, six found that adolescents were comparing themselves more frequently to peers, celebrities and influencers. The studies consistently reported increased experiences of body dissatisfaction (Papageorgiou et al. 2022a; Burnette et al. 2017; Artigues‐Barberà et al. 2025; Mahon and Hevey 2021), and experimentation with weight control behaviours in their participants (Ando et al. 2021; Spurr et al. 2013).

Papageorgiou et al. (2022a) reported negative appearance comparisons when viewing pictures on SM, stating that 100% of the adolescent girls (24) that participated identified body image as a ‘major concern’, reporting increased levels of insecurity and self‐consciousness about their appearance when using SM platforms such as Instagram, in particular. While it was consistently reported that viewing images of both celebrities and peers exacerbated comparisons, participants noted that viewing pictures posted by their peers exerted a greater negative influence, suggesting that this was because their peers represented a more ‘realistic’ standard for how young girls should look than that of celebrities.

I guess, you know they're celebrities, so something must have gone into it [a photo]. It's not just a photo, but I think if it's someone you know or someone your age, it's like, “Wow, that really could be me,” or “People my age are looking like this or doing this kind of stuff.” So, I think it would have a worse effect. (Papageorgiou et al. 2022a)

When I see girls my age [on Instagram], I just compare myself to them ‘cause I know it's kind of reality, if that makes sense, to know that someone my age can look like that and then why don't I look like that? (Papageorgiou et al. 2022a)

It must be considered that this study was conducted on a sample of 24 adolescent girls from a non‐government school, risking sampling bias. This limits the generalisability of the research to wider contexts, as the experiences of adolescents from a lower socioeconomic background may differ due to decreased access to digital technology, and therefore SM. Additionally, the use of an all‐female sample may limit the depth and diversity of the perspectives captured by the study, which is particularly important when investigating complex lived experiences, such as the aim of this review.

A focus group study conducted by Spurr et al. (2013) found that adolescents reported images viewed on SM as influencing body dissatisfaction and the development of disordered eating behaviours in their sample of 46 adolescents. One participant described that seeing models and celebrities online made her feel self‐conscious due to the physical incongruences between them, causing her to ‘stop eating’ and ‘try a bunch of different diets’. Both male and female participants expressed a perception that SM negatively affected their perceived body image, which, for some, led to harmful weight control behaviours.

### 4.3 Societal Standards and Pressures

Five of the seven included studies found that SM imposes unrealistic and unattainable standards commonly internalised by adolescent users. The studies' findings highlighted harmful behaviours such as self‐objectification (Ando et al. 2021; Papageorgiou et al. 2022b; Artigues‐Barberà et al. 2025) and negative self‐perceptions as a result (Spurr et al. 2013).

Ando et al. (2021) conducted an interview study with 29 Japanese adolescents, discovering the perception that SM has facilitated the globalisation of the ‘Western ideal’ body type, negatively influencing beauty standards across cultures. The participants frequently reported the influence of the West as an external source of beauty ideals, most of which embody extreme thinness. Participants consistently reported that they felt pressure towards thinness on SM, leading to weight control behaviours and negative self‐perceptions. Similarly, participants in Spurr et al. (2013) focus group study reported pressures to conform to the ‘Western ideal’, resulting in ‘feelings of discomfort in their own body’. In contrast, Papageorgiou et al. (2022b) found that some participants did not perceive this increased focus on women's bodies on SM as harmful and viewed it as normal.

### 4.4 Validation

Four included studies found that validation plays a large role in shaping adolescents' online behaviour and self‐presentation. The studies' findings indicated that this performative engagement distorts self‐expression and identity (Burnette et al. 2017; Artigues‐Barberà et al. 2025) and reinforces appearance‐based self‐worth, leading to self‐objectification and emotional distress (Papageorgiou et al. 2022a, 2022b).

An interview study by Papageorgiou et al. (2022a) found that adolescents often equate SM engagement metrics with social approval. Positive validation in the form of likes and compliments was found to temporarily boost self‐esteem and feelings of social belonging, leading adolescents to post selectively to gain social approval. Additionally, Burnette et al. (2017) and Papageorgiou et al. (2022b) both found that participants reported posting images that were more sexualised on SM to gain approval from their peers through likes and comments. Papageorgiou et al. (2022b) also concluded that societal norms encourage female adolescents to post sexualised content to receive more likes and comments from their peers. The study found that this reinforces the message that adolescents' value is based on their appearance, leading to participants feeling pressured to conform to societal norms that ‘reward’ adolescent girls for posting sexualised images.

That's why they do it, because they know they're gonna get more likes and that's really bad that that's what they have to do. (Papageorgiou et al. 2022b)

Unrealistic beauty and body standards is the worst thing. I don't really like having those high standards to measure up to, especially when I know they are pretty unrealistic. (Papageorgiou et al. 2022b)

However, this was not the case for the whole participant group, as all of the studies found that some participants viewed this behaviour as “attention‐seeking” and did not participate;

I personally would never do that, just because I don't want that attention, but if they want it, then go ahead. (Papageorgiou et al. 2022b)

### 4.5 Theme 2: Gendered Experiences

Within the included studies, adolescents commonly described SM as detrimental to their mental health; however, some studies reported perceptions that the effects are gender specific (Spurr et al. 2013; Papageorgiou et al. 2022b; Artigues‐Barberà et al. 2025; Mahon and Hevey 2021). Sub‐themes were identified as gender differences and media literacy.

### 4.6 Gender Differences

Of the seven included studies, three reported that SM exerted varying effects on their participants dependent on gender (Spurr et al. 2013; Artigues‐Barberà et al. 2025; Mahon and Hevey 2021). Papageorgiou et al. (2022b) also highlighted potential gender differences, although they only included females within their research. The main findings within this were that female participants were less likely to seek out positive content than their male counterparts (Mahon and Hevey 2021) and were more likely to internalise unattainable beauty standards (Spurr et al. 2013; Papageorgiou et al. 2022b).

Spurr et al. (2013) and Mahon and Hevey (2021) presented this discussion in the most detail, as both studies included male and female participants. Both studies found that male and female participants were influenced by the notion of an ‘ideal’ body type, determining that this ideal is ‘lean and muscular’ for males and ‘thin’ for females. A key finding indicated that certain male participants perceived SM as a motivating environment, regarding these idealised bodies as ‘attainable through hard work and effort’ (Mahon and Hevey 2021). One participant expressed that the bodies he encountered online ‘motivated him to go to the gym and be more disciplined’ (Spurr et al. 2013). Moreover, one study revealed that female participants often regarded idealised bodies as unattainable, resulting in increased levels of appearance‐based comparisons and body dissatisfaction compared to their male counterparts (Spurr et al. 2013). Conversely, several male and female participants from both studies expressed that exposure to these idealised bodies on SM contributed to self‐criticism and body dissatisfaction. One male participant reported that ‘boys are negatively affected by this stuff just as much as girls’. Artigues‐Barberà et al. (2025) demonstrated similar findings, stating that the negative effects of SM are becoming a ‘rising issue in young men’.

### 4.7 Media Literacy

All of the included studies highlighted the importance of media literacy in mitigating the negative effects of unrealistic beauty standards and harmful influences perpetuated by SM in their findings (Papageorgiou et al. 2022a, 2022b; Burnette et al. 2017; Spurr et al. 2013; Ando et al. 2021; Artigues‐Barberà et al. 2025; Mahon and Hevey 2021).

In Burnette et al. (2017) focus group study, several participants endorsed social comparisons and appearance concerns as a result of viewing pictures of both celebrities and peers on SM. However, participants were able to identify the negative consequences that this had on their body image and had an awareness of the artificial nature of SM. Five of the six focus groups demonstrated high media literacy by being able to recognise the edited and retouched nature of images, in particular celebrities', posted on SM, with one participant expressing that she doesn't compare herself to celebrities because ‘they live in a different world, with makeup artists and Photoshop’.

This correlates with the findings of Papageorgiou et al. (2022a) and Papageorgiou et al. (2022b), in which the participant groups recognised the unhelpful culture on SM, that ‘your value is based on your appearance’, and were able to report strategies such as ‘Protective Filtering’ that they employed to prevent self‐comparison from this. This involves ‘internalising messages that preserve positive body image and rejecting ones that are potentially harmful’ (Burnette et al. 2017). Mahon and Hevey (2021) also found that media literacy was high among their participants, but particularly in females, with participants reporting that ‘if [she] used filters, [she] would look way better’, and ‘know[ing] that they [celebrities/social influencers] are photoshopped’ aided in psychologically distancing oneself from perceived pressures. They found that male participants were, on average, less aware of photo manipulation and editing, and reported that the bodies they viewed on SM were attainable with hard work. This echoes the ideas presented in the previous sub‐theme, that SM is a more motivating space for men.

### 4.8 Theme 3: Protective Roles and External Influences

Participants in six of the included studies reported that education and support in school and home environments play a crucial role in the promotion of positive body image in their participants (Papageorgiou et al. 2022a, 2022b; Burnette et al. 2017; Artigues‐Barberà et al. 2025; Mahon and Hevey 2021). There was also a reported perception that body‐positive content on SM had a positive influence on participants in some studies (Ando et al. 2021), but not in others (Mahon and Hevey 2021).

### 4.9 Role of Schools and Parents

Burnette et al. (2017) interview study found that participants viewed their school and home environments as vital in mitigating the negative impacts that SM had on their body image. They reported that their school fostered media literacy, critical thinking, and body positivity through curricula that challenged unrealistic beauty standards and promoted self‐acceptance. The sample also indicated that their school environment was a ‘unique culture’ and was much more supportive than those of their peers, with one participant stating that their school ‘just care a lot more’, and another reporting that ‘they teach you how to like yourself’. Artigues‐Barbera et al. (2025) and Ando et al. (2021) found that the discrepancy between how schools approach body image education invokes a further negative effect on adolescents. Both studies reported that bullying, lack of teacher awareness and peer dynamics exacerbated body dissatisfaction in their participants.

Parents reinforce positive body image by modelling healthy self‐esteem, fostering open communication, and discouraging appearance‐based validation (Burnette et al. 2017). Ando et al. (2021) found that the absence of positive parental influence further increased negative self‐perceptions, with one participant reporting that she received pressure from her family:

my mom sometimes goes “you should lose some weight” and I say, “I know, I know”

This demonstrates how adolescents can feel explicit pressure to lose weight and engage in dieting behaviours from caregivers, in turn causing them to internalise the idea that ‘skinny is the best’ (Ando et al. 2021).

### 4.10 Promotion of Positive Body Image

Five included studies highlighted the external and internal strategies used by adolescents to promote a positive body image, both on and off SM. These include engaging with body‐positive content online (Ando et al. 2021) and use of protective filtering (Burnette et al. 2017). Participants from Burnette et al. (2017) study expressed that engaging in protective filtering empowered them to shape their own body image and to appreciate the qualities of their peers without the sense of jealousy that can often lead to self‐comparison. The researchers suggested that this indicated enhanced media literacy. Papageorgiou et al. (2022a) and Mahon and Hevey (2021) similarly found that protective filtering techniques proved useful to reduce appearance comparisons and uphold positive body image. Ando et al. (2021) participants indicated that body positive campaigns on SM, such as ‘Savage X Fenty, Dove and Calvin Klein’ that present diverse bodies, promote a healthier perception of body image. Conversely, Mahon and Hevey (2021) found that female participants were negatively affected by all SM content regarding body image, regardless of whether this is positive, with one female participant expressing that, despite the presence of body positive content existing, content including ‘skinnier ones [individuals]’ was observed to ‘get more positivity back than the plus size ones [individuals] would’.

## 5 Discussion

In all seven included studies, adolescents described SM use as having influenced their perceived body image. This included increased self‐comparison, implementation of harmful societal standards and pressures, and increased reliance on validation from others. An increase in self‐comparison to both peers and celebrities was the most commonly reported as influencing perceived body image (Papageorgiou et al. 2022a; Burnette et al. 2017; Ando et al. 2021; Spurr et al. 2013; Artigues‐Barberà et al. 2025; Mahon and Hevey 2021). Papageorgiou et al. (2022a) and Spurr et al. (2013) reported increased body dissatisfaction due to negative appearance comparisons when viewing photos of others on SM, which aligns with Rodgers et al. (2020) and Holland and Tiggeman's (2016) studies showing that adolescents, particularly females, internalise societal beauty norms, increasing their risk of body dissatisfaction and disordered eating behaviours. Spurr et al. (2013) also considered the additional effects of body dissatisfaction on participants, exploring the development of disordered eating and weight‐control behaviours, a finding echoed by Ando et al. (2021).

Tiggemann (2011) explains this using Sociocultural Theory, proposing that the dominance of the unrealistic Western ideal in SM encourages women to internalise and aspire to emulate these despite their impossibility. Thompson and Stice (2001) describe this as the internalisation of the ‘thin ideal’ as individuals accept dominant societal ideals of attractiveness in response to the way in which these ideals have been approved and validated by others. As individuals invariably fail to achieve this, body dissatisfaction and disordered eating are invoked (Tiggemann 2011; Bair et al. 2012).

A systematic review by Vincente‐Benito and Ramirez‐Duran (2023) found that sociocultural influences perpetuated by SM, such as socially accepted ideals of appearance, were instrumental in the development of mental health problems among adolescents. Concerns regarding appearance can lead to a reduced sense of connectedness and relatedness, potentially further exacerbating the impact of SM on mental health; however, professional support can act as a protective factor (Guimond et al. 2025). Mental health nursing is inherently relational, and it may be that the therapeutic relationship is integral to mitigating the impacts of SM by fostering the development of an alliance wherein the adolescent feels fundamentally validated and understood, without a primacy being placed on their appearance (Hartley et al. 2020).

Another key finding explored in four of the included studies was the presence of societal standards and pressures on SM, which lead to self‐objectification (Ando et al. 2021; Papageorgiou et al. 2022b; Artigues‐Barberà et al. 2025). Objectification theory (Fredrickson and Roberts 1997; Opara and Santos 2019) proposes that people gradually beA0;al. 2020).

Another key finding explored in four of the included studies was the presence of societal standards and pressures on SM, which lead to self‐objectification (Ando et al. 2021; Papageorgiou et al. 2022b; Artigues‐Barberà et al. 2025). Objectification theory (Fredrickson and Roberts 1997; Opara and Santos 2019) proposes that people gradually begin to view themselves as objects which can be evaluated primarily on the basis of their appearance and therefore develop an increased reliance on external validation (Burnette et al. 2017). Participants reported that validation through likes and comments can foster the notion that self‐worth is contingent on appearance, reinforcing the need for external approval. This prompted participants in the studies by Papageorgiou et al. (2022b) and Burnette et al. (2017) to post an increasing amount of sexualised content. Given that Erikson's (1968) theory of identity development conceptualises adolescence as a vital period of identity formation and increased sensitivity to peer evaluation, the participants' posting of this sexualised content may represent an attempt to achieve social acceptance and conform to their perceived appearance and gender norms. Consistent with this perspective, Vandenbosch and Eggermont's (2016) meta‐analysis indicated that SM use amplifies exposure to narrowly defined appearance standards, suggesting that a thin body for girls and a muscular body for boys are deemed necessary for sexual attractiveness. In this context, personality becomes secondary to appearance‐based judgements, influencing adolescents' self‐concept and shaping their perceptions of what aspects are deemed important by society. Over time, these factors can contribute to low self‐esteem, anxiety, and disordered eating behaviours (Holland and Tiggeman 2016), highlighting SM's critical influence on body image during adolescence.

The factors influencing perception of body image can be viewed in the context of an intersectional theoretical framework, understanding that adolescents may be subjected to multiple factors impacting perception of body image such as gender, ethnicity, sexual orientation, and sociocultural status (Opara and Santos 2019). Therefore, consideration should be given to the ecological systems from which the participants were recruited. Several studies recruited from private schools (Papageorgiou et al. 2022a, 2022b; Burnette et al. 2017; Mahon and Hevey 2021). Despite there being limited evidence to suggest that attending a private school impacts mental health, private schools often have more resources than public schools to support mental health (Henderson et al. 2022). Consequently, consideration should be given to potential variations in experiences between state and privately funded school environments.

Some included studies identified in this review specifically reported the adverse influence of SM on adolescents' formulation of identity and self‐perception being gender specific. This theme discusses the different experiences of SM by male and female adolescents, concerning varying levels of media literacy, and the different appearance ideals placed on each gender. Spurr et al. (2013) and Mahon and Hevey (2021) presented this discussion in the most detail, due to their participant groups containing both male and female adolescents. As discussed in the previous theme, both studies found parallels between male and female participants' understanding of an ideal body type perpetuated on SM. However, similar to the findings of Hargreaves and Tiggemann (2004), the ideal for females was based on thinness, and the ideal for males was a lean and muscular build. The studies additionally highlighted that both male and female participants experienced increased self‐criticism and body dissatisfaction as a result (Spurr et al. 2013; Mahon and Hevey 2021). These findings are consistent with Stratton et al. (2015) study, which found that sociocultural factors such as media, peers and parents influence the development of body dissatisfaction in male adolescents. More specifically, it has been suggested that the Western ideal of lean muscularity exacerbates male body dissatisfaction to a similar extent to which the thin ideal influences girls (Murray et al. 2018). These comments support the conclusion that, while the ideal body type portrayed on SM is dependent on gender, adolescent body image is suffering collectively as a result of the idealised media ecology which is exacerbated by SM.

Several of the included studies reported high levels of media literacy among their participants, particularly females. Burnette et al. (2017) and Mahon and Hevey (2021) found that female participants were more aware of the artificial nature of SM and could employ protective techniques to reject harmful messages more frequently than their male counterparts. Additionally, Vincente‐Benito and Ramirez‐Duran (2023) indicated that a low level of media literacy among the samples in their systematic review was a key determinant of body image. While the literature suggests varied levels of media literacy across adolescent populations, it is undeniable that gaining these skills may help to minimise the harmful impact that SM has on body image. Geraee et al. (2015) reported a statistically significant (*p* = 0.001) increase in adolescents' mean knowledge scores after implementing a media literacy training program, improving critical reflection skills in response to harmful media messages. A similar workshop showed a positive effect on participants' media literacy after only 1 day (Kupersmidt and Scull 2011). Mental health nurses need to be mindful of not only the threats posed by SM but also the ways to mitigate these threats through interventions, such as promoting media literacy. Nurses are often well placed, due to the quality of the therapeutic relationship and ability to critically engage with evidence, to support adolescents in developing. However, for such interventions to be effective, the health professionals involved need to also have a suitable level of media and digital literacy (Amin et al. 2025).

The final theme will explore the protective roles that schools and parents play in promoting a positive body image to mitigate the harmful effects of SM, as discussed in the previous themes. Some of the included studies found that education and support from school and home environments exerted a positive influence by fostering self‐acceptance (Burnette et al. 2017). Similarly, Ando et al. (2021) discovered that the absence of this external support exacerbated body dissatisfaction. Tort‐Nasarre et al. (2023) found that parents and educators directly influenced how participants perceived their self‐image in their interview study. They reported that providing a safe environment and encouraging communication helped adolescents to build a healthy self‐image and improve their self‐esteem. The researchers further stated that educators are responsible for providing media education to encourage adolescents to critically respond to negative messages on SM. Sundgot‐Borgen et al. (2018) conducted one of the first randomised controlled intervention studies aimed at promoting positive body image in a Norwegian school environment. The researchers collaborated with educators to implement a Healthy Body Image programme, which demonstrated effectiveness in reducing comparisons and enhancing media literacy within a school setting. This reinforces the conclusions made by Tort‐Nasarre et al. (2023), suggesting that adolescents' positive experiences related to body image cannot be fully understood without acknowledging the significant roles played by families and educators. These findings further underscore the importance of implementing interventions in school and home environments to enhance self‐esteem, broaden perceptions of beauty standards, and facilitate open dialogue between adolescents and their parents and educators.

### 5.1 Limitations

Whilst the search undertaken as part of this review was comprehensive, the literature search was confined to three databases, which may have excluded wider sources. Additional databases and grey literature may be able to contribute wider sources to the review. The heterogeneity between the studies provides an international and cross‐cultural insight into the perceived impact of SM. However, due to the focus on lived experience, consideration does need to be given to the ecological environments from which participants were recruited and potential intersectional impacts which might ameliorate or exacerbate the perceived impacts of SM. For example, several of the participant samples were from affluent, non‐government school populations, and their lived experiences may not be reflective of adolescents in less affluent environments. Whilst potential gender differences have been identified, only three of the seven studies included in this review contained male participants, which limits the strength of gender comparisons.

## 6 Conclusion

This review examined adolescents' perceptions and lived experiences of the relationship between SM use and body image, while also identifying protective factors, namely supportive school and home environments, that help to mitigate perceived effects. The findings underscore the need for further empirical research exploring how SM can be used to promote body positivity and self‐acceptance, particularly through the inclusion of diverse and realistic representations. Such insights could inform the development of school‐based curricula and adolescent mental health services within the UK. While there is a definitive need for further research to be conducted in this area, the conclusions drawn from this review are well substantiated by existing evidence.

Overall, this review highlights that adolescents are reporting lived experiences of body dissatisfaction and appearance‐related concerns. These issues are closely associated with elevated incidences of disordered eating behaviours among this population, because of the self‐comparison, societal pressures and self‐objectification that is supercharged through SM use. Nevertheless, emerging evidence suggests the potential for meaningful change. Recent developments, such as body‐positive SM campaigns and the prospective incorporation of compassion‐focused interventions to improve body image within educational settings, indicate encouraging prospects for intervention that warrant continued exploration and evaluation.

## 7 Relevance for Clinical Practice

The findings of this review highlight the perception of adolescents that SM negatively affects body image in various ways. These impacts include self‐objectification, body dissatisfaction, self‐criticism, and disordered eating behaviours. The emerging themes suggest a series of implications for both research and practice. Theme 1 highlights the perceived influence of SM on both identity and self‐perception. Although these issues were prevalent among the participant samples explored, research has also highlighted the potential for change. Mahon and Hevey (2021) and Burnette et al. (2017) demonstrated that implementing compassion‐focused education in school environments significantly aided in mitigating these negative effects, promoting body positivity and self‐acceptance in their participants.

Theme 2 revealed the potential for gender‐specific differences in the perceived impact of SM on mental health and highlighted the importance of media literacy among adolescents. The results from this review further emphasise the importance of supporting adolescents to develop media literacy and critical thinking (Ku et al. 2019) to mitigate the potentially pernicious impact of SM on perceived body image.

Theme 3 identified the protective roles and influence of families, teachers and health professionals. To ensure that future healthcare professionals are adequately equipped and knowledgeable, they need to be aware of the potentially damaging effects of SM on adolescents and be able to provide effective interventions to practice in line with the most current available evidence (Nursing and Midwifery Council 2018; Vincente‐Benito and Ramirez‐Duran 2023). Pre‐registration nursing curriculums must reflect this need alongside additional training sessions for healthcare workers of all levels to enhance their scope of practice when delivering care to adolescents.

## Funding

The authors have nothing to report.

## Conflicts of Interest

The authors declare no conflicts of interest.

## Data Availability

The authors have nothing to report.

## Appendix A

Included studies matrix

**Table:**

Study	Country of origin	Sample	Phenomenon	Data collection method	Outcome
Papageorgiou et al. (2022a)	Australia	24 adolescent girls	Social media effects on body image	One‐on‐one semi‐structured interviews	Comparison, validation, expectation‐ self‐objectification is a prominent issue. Influence of social media on body image was perceived by participants as negative. Participants found it difficult to name positive influences on social media. Participants had high media literacy, and were able to suggest how the experiences of comparison on social media led to negative feelings, and were able to suggest strategies to mitigate this. Participants were able to show critical thinking when viewing images of celebrities. Comparison to peers facilitated stronger negative beliefs, due to peers being perceived as more relatable and realistic than celebrities. Protective filtering and positive body image identified as useful to uphold positive body image by participants. Participants suggests that body dissatisfaction as a result of social media could be mental state and mood‐based‐ girls with higher tendency to internalise appearance ideals show higher levels of negative appearance comparisons. Gaining approval and validation on social media through likes and comments was frequently mentioned by participants. Instagram and showing likes. Role of schools and parents in promoting positive body image‐ both can play protective roles.
Burnette et al. (2017)	USA	38 adolescent girls	Social media effects on body image	Questionnaires and focus groups	Social media use was high in the studied age group, with 90% of participants reporting daily use, and 50% reporting visiting sites multiple times a day. Popularity with early adolescents lies in highly visual social media platforms such as Instagram, Snapchat and TikTok. Participants reported that the use of photo‐based platforms leads to appearance comparison, and body dissatisfaction. Parental influences/restrictions affect social media use in participants. While participants expressed displeasure with parents restricting social media use, it is an effective mediator for social media use. Media literacy is high among adolescents. Participants demonstrated attitudes and strategies to mitigate body dissatisfaction, such as protective filtering. Sample were able to recognise the artificial and retouched nature of images online, particularly celebrities. Only some participants endorsed social media leading to appearance comparisons and body image issues‐ social desirability leads to biased/censored responses? School environment important‐ ‘fat talk’, body image clubs.
Spurr et al. (2013)	Canada	46 adolescents	Social media effects on body image	Focus groups	Participants were able to understand that the images they see on social media were idealised and unrealistic, but they still have a profoundly negative effect on self‐image. Facilitation of self‐comparison, leading to negative feelings of self, and disordered eating behaviours. Male participants were negatively affected as well as female participants, however they felt that the bodies they see online motivated them to go to the gym more, and be more disciplined‐ social media is a more motivating space for males. Discomfort with their body, and can see this developing from a young age due to societal values‐ if you are pretty, you are more liked. Pressure to conform to the “Western ideal body”‐ negative associations with being ‘fat’. Influencers and celebrities with the ideal body caused participants to self‐objectify, and focus on the parts of themselves that were incongruent with the person they were seeing. Female participants stated that a girl would be ‘weird’ if she did not dislike at least one thing about her body/appearance. Male participants idealised a lean and muscular build, whereas females idealised a thin build. Less prevalence of negative body image experienced by male participants‐ exploring gender differences.
Ando et al. (2021)	Japan	29 adolescent girls	Social media effects on body image and eating behaviour	Group and individual semi‐structured interviews	Pressure towards thinness on social media and idealisation of the Western ideal has led to weight control behaviours, experimentation with dieting, and negative perceptions of the self. Social media facilitates globalisation, which influences beauty ideals cross‐culturally. Pressure towards plastic surgery increased by pressure towards conformity to the ideal standard. Influence of the US/West was heavily mentioned by the participants as an outside source of beauty ideals, most of whom embody extreme thinness and other particular features, such as enhanced lips. Some participants reported that the American standard placed less emphasis on thinness than the Japanese standards. Participants mentioned certain campaigns such as Savage × Fenty, Dove and Calvin Klein as producing content with more diverse bodies, promoting a healthier perception of body image. Bullying and pressure in school environments contributes to negative self‐perceptions. Family environments contribute, with one participant reporting pressure from her family to lose weight and engage in dieting behaviours. Participants expressed understanding and criticism of the pressures imposed on them by social media, describing them as unrealistic and unattainable. However, we can see that they still have a profound negative effect. Japanese men source of pressure.
Papageorgiou et al. (2022b)	Australia	24 adolescent girls	Social media, sexualised images and their effects on body image	One‐on‐one semi structured interviews	Participants had an awareness of the sexualisation of women and girls on social media‐ skewed gender norms. Focus on female bodies, sexual objectification, normalisation of this on social media. Participants were able to recognise this, but did not necessarily view it as harmful‐ they described people in their age group (and themselves) posting sexualised images regularly, and view this regularly on social media from celebrities, so they perceive it as normal. Gaining validation from likes and comments plays a key role in exacerbating norms. Society ‘supports’ girls into doing it, in that they receive more likes when they do. Conforming with peer pressure‐ peer roles and comparisons. Value based on appearance‐ self‐objectification. Unrealistic body standards perpetuated by celebrities. Participants were able to recognise that this was unhelpful‐ high media literacy. Responsibilities of schools and parents to educate adolescents on risks and harms of social media‐ participants suggested that “telling girls what to do” as opposed to offering harm minimisation strategies was unhelpful. Some participants had received education about dangers from their school, but others hadn't.
Artigues‐Barberà et al. (2025)	Spain	9 adolescents, 7 parents, 8 school teachers	Factors in supporting adolescents in achieving positive body image, with a focus on social media being the main barrier to this	Semi‐structured interviews	Society promotes unattainable beauty standards that adolescents internalise, leading to negative perceptions of the self. Teachers suggested that this has a profound effect, but that adolescents are unaware of this. They also suggested that adolescents need spaces to feel heard and understood‐ home dynamics/school environment is important. Social media facilitates comparison with others based on unattainable beauty standards, increasing body image concerns and body dissatisfaction. Social rejection decreases self‐esteem. Adolescents were more likely to dress and act similarly to their peers to prevent this. Male and female participants were negatively affected, this is becoming a rising issue with boys. Lack of family communication, unfavourable family environments, lack of teacher awareness/training are barriers to promoting positive body image in adolescents. Female participants were more affected by comments made by male peers, is this contributed to social media as well? Thin women are valued more on social media, which is internalised by adolescents.
Mahon and Hevey (2021)	Ireland	29 adolescents‐ 23 female, 6 male	Social media effects on body image	Qualitative focus groups	Female participants in the study suggested that social media negatively impacted their body image. Appearance‐focused platforms such as Instagram facilitated comparisons with peers, celebrities and influencers, negatively impacting body image, causing body dissatisfaction. Validation from likes and comments caused female participants to feel pressure when uploading pictures of themselves. Male participants tended to seek out positive content, whereas female participants tended to take a passive role and “put up” with harmful content‐ normalisation, gender differences. Female participants reported that even body positive content had a negative effect on them‐ any content related to body image. Female participants had an awareness that the bodies they see on social media are sometimes unrealistic, and are unattainable. Limiting use of social media, and avoiding, unfollowing and ignoring problematic content seemed to be popular among participants. Participants exhibited aspects of protective filtering to reduce comparisons. High media literacy present, but found to not always be helpful, as participants still struggled to accept their own bodies. Participants reported self‐criticism, self‐comparison and body dissatisfaction as a result of social media. Male participants less aware of photo manipulation and editing on social media, and tended to view any physique as attainable with hard work and effort‐ social media is a less pressurising and more motivating for boys. Compassion‐focused approaches are more suitable to mitigate body dissatisfaction.

## Appendix B

Critical appraisal of included studies using CASP appraisal tool. As per critical skills programme (2022)

**Table:**

Reference	Was there a clear statement of the aims of the research?	Is a qualitative methodology appropriate?	Was the research design appropriate to address the aims of the research?	Was the recruitment strategy appropriate to the aims of the research?	Was the data collected in a way that addressed the research issue?	Has the relationship between researcher and participant been adequately considered?	Have ethical issues been considered?	Was the data analysis sufficiently rigorous?	Is there a clear statement of findings?	Is the research valuable?	Additional comments
Papageorgiou et al. (2022a)	Yes	Yes	Yes	Yes	Yes	Limited	Yes	Yes	Yes	Yes	Female only participants. Researcher bias was not considered.
Burnette et al. (2017)	Yes	Yes	Yes	Yes	Yes	Yes	No	Yes	Yes	Limited	Sample size of girls from private school only.
Spurr et al. (2013)	Limited	Yes	Yes	Limited	Yes	No	Yes	Yes	Yes	Yes	Aim not clearly stated. Recruitment of participants not defined extensively. Mixed sample of female (*n* = 14) and male (*n* = 4). Researcher bias not considered.
Ando et al. (2021)	Yes	Yes	Yes	Yes	Yes	Yes	No	Yes	Yes	Yes	Ethical considerations not reported. Sample includes older adolescents termed ‘young women’.
Papageorgiou et al. (2022b)	Yes	Yes	Yes	Yes	Yes	Yes	Yes	Yes	Yes	Yes	Small, female only sample size. Recruitment from non‐government schools and youth research council.
Artigues‐Barberà et al. (2025)	Yes	Yes	Yes	Yes	Yes	Yes	yes	Yes	Yes	Yes	Small sample size of adolescents, mixed of female (*n* = 7) and male (*n* = 2) alongside parents and teachers.
Mahon and Hevey (2021)	Yes	Yes	Yes	Yes	Yes	Yes	Yes	Yes	Yes	Yes	Mix of male (*n* = 6) and female (*n* = 23) participants. One ‘urban private school’ and one ‘rural community school’

## Appendix C

Identification of themes

**Table:**

	Identity and self‐perception			Gendered experiences		Protective roles and external influences	
	Self‐comparison	Societal standards/pressures	Validation	Gender differences	Media literacy	Role of schools and parents	Promotion of positive body image
Papageorgiou et al. (2022a)	X		X		X	X	X
Burnette et al. (2017)	X		X		X	X	X
Spurr et al. (2013)	X	X		X	X		
Ando et al. (2021)	X	X			X	X	X
Papageorgiou et al. (2022b)		X	X	X	X	X	
Artigues‐Barberà et al. (2025)	X	X	X	X	X	X	X
Mahon and Hevey (2021)	X	X		X	X	X	X
